# 887. Implementation of Long-acting Injectable Cabotegravir/Rilpivirine for HIV-1 Treatment at a Ryan White-funded Clinic in the U.S. South

**DOI:** 10.1093/ofid/ofab466.1082

**Published:** 2021-12-04

**Authors:** Lauren F Collins, Lauren F Collins, Della Corbin-Johnson, Meron Asrat, Tonya Rankins, Latoya Harrison, Alton Condra, Jeri Sumitani, Bradley L Smith, Wendy Armstrong, Wendy Armstrong, Jonathan Colasanti

**Affiliations:** 1 Division of Infectious Diseases, Emory University School of Medicine, Atlanta, GA; 2 Grady Healthcare System, Infectious Diseases Program, Atlanta, GA; 3 Grady Health System, Decatur, GA; 4 Emory University, Atlanta, GA

## Abstract

**Background:**

In January 2021, the first ever long-acting injectable (LAI) antiretroviral therapy (ART), cabotegravir/rilpivirine (CAB/RPV), was approved for maintenance HIV-1 treatment in select patients with virologic suppression. LAI-ART has the potential to improve ART adherence, reduce HIV stigma, and promote equity in care outcomes, however, implementation in real-world settings has yet to be evaluated.

**Methods:**

We launched a pilot LAI-ART program at the largest Ryan White-funded HIV clinic in the Southeast. From 4/14/21 to 5/14/21, providers referred patients interested and willing to switch to LAI-CAB/RPV who met screening criteria. Our interdisciplinary LAI team (Clinician-Pharmacy-Nursing) verified clinical eligibility (HIV-1 < 200 c/ml ≥6 months and no history of virologic failure, resistance to either drug, or chronic HBV infection) and pursued medication access for 28-day oral lead-in and monthly injectable CAB/RPV. We describe demographic and clinical variables of referred PWH and early outcomes in accessing LAI-ART.

**Results:**

Among 42 referrals, median age was 40.5 (Q1-Q3, 32-52) years, 83% were men, and 76% Black. Payor source distribution was 26% Private, 19% Medicare, 10% Medicaid, and 45% ADAP. At the time of referral, median CD4 count was 583 (Q1-Q3, 422-742) cells/mm^3^ and median sustained HIV-1 RNA < 200 c/ml was 1427 (Q1-Q3, 961-2534) days. A total of 35 patients (74%) met clinical eligibility for LAI-CAB/RPV, including 4 patients who required a transition off proton pump inhibitor therapy to accommodate oral RPV. Ineligible PWH were excluded due to evidence of RPV resistance (n=5), possible RPV hypersensitivity (n=1), and HIV non-suppression (n=1). The table summarizes the process of pursuing LAI-ART access for the initial 10 enrollees by insurance status.

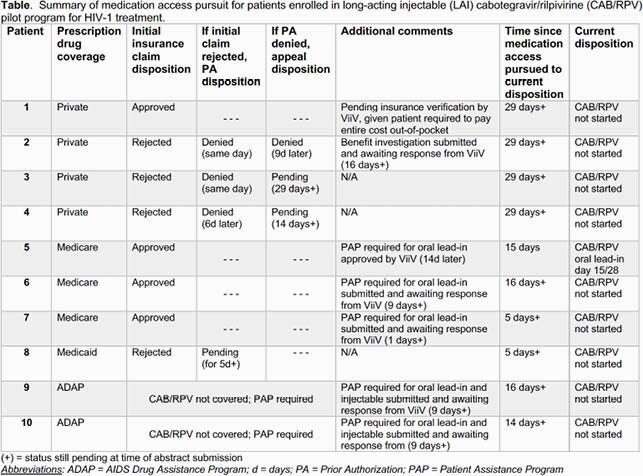

**Conclusion:**

Our experience implementing LAI-ART at a Ryan White-funded HIV clinic in the Southern U.S. has been challenged by substantial human resource capital to attain drug, delayed therapy initiation due to insurance denials, and patient ineligibility primarily due to concern for potential RPV resistance. These barriers may perpetuate disparities in ART access and virologic suppression among PWH and need to be urgently addressed so that LAI-ART can be offered equitably.

**Disclosures:**

**Lauren F. Collins, MD, MSc**, Nothing to disclose **Bradley L. Smith, Pharm.D., AAHIVP**, **Gilead Sciences, Inc** (Advisor or Review Panel member) **Wendy Armstrong, MD**, Nothing to disclose **Jonathan Colasanti, MD**, **Integritas CME** (Consultant, develop and deliver CME content around Rapid Entry/Rapid ART)

